# Should We Intervene Early in Asymptomatic Aortic Stenosis? Evidence From a Meta-Analysis

**DOI:** 10.1097/MJT.0000000000001881

**Published:** 2025-02-07

**Authors:** Saad Ahmed Waqas, Muhammad Saad, Haiqa Aamer, Muhammad Umer Sohail, Muhammad Rashid, Raheel Ahmed

**Affiliations:** 1 Department of Medicine, Dow University of Health Sciences, Karachi, Pakistan;; 2 Keele Cardiovascular Research Group, Centre for Prognosis Research, Institute for Primary Care and Health Sciences, Keele University, Keele, United Kingdom;; 3 Department of Cardiovascular Sciences, Glenfield Hospital, University Hospitals of Leicester NHS Trust, Leicester, United Kingdom;; 4 NIHR Leicester Biomedical Research Centre, University of Leicester, Leicester, United Kingdom; and; 5 National Heart and Lung Institute, Imperial College London, London, United Kingdom.

## Abstract

Supplemental Digital Content is Available in the Text.

***To the Editor:***

Aortic stenosis (AS) is the most prevalent valvular heart disease in developed nations, with a rising incidence in aging populations.^1^ For symptomatic patients with severe AS, current American Colllege of Cardiology, American Heart Association, and European Society of Cardiology guidelines assign a class I recommendation for aortic valve replacement—either surgical (SAVR) or transcatheter (TAVR)—to alleviate symptoms and enhance survival.^2,3^ However, the optimal timing of intervention for asymptomatic patients with severe AS remains a subject of active debate. Although guidelines suggest postponing intervention until the onset of AS-related symptoms or left ventricular systolic dysfunction, recent meta-analyses have highlighted improved outcomes with early intervention in such patients.^4–6^ However, these prior analyses largely relied on nonrandomized studies, which may limit their robustness. To address this gap, our study is the first meta-analysis to exclusively include randomized controlled trials (RCTs), incorporating 2 recent trials—EARLY TAVR and EVOLVED—that were previously unavailable. By focusing on RCTs, our study aims to provide a more definitive assessment of the impact of early intervention versus conservative management on key outcomes in asymptomatic severe AS.

A comprehensive search of PubMed, Scopus, and Cochrane databases was conducted from inception through October 2024 without language restrictions. Studies were included if they (1) were RCTs or their post hoc analyses and (2) enrolled patients with asymptomatic severe AS randomized to early intervention—with either SAVR or TAVR—versus conservative management. Key outcomes analyzed were all-cause mortality, cardiovascular (CV) mortality, hospitalization for heart failure (HHF), myocardial infarction, sudden cardiac death (SCD), and stroke. For each outcome, hazard ratios (HRs) or risk ratios (RRs) with 95% confidence intervals were extracted and pooled using a random-effects model: the inverse variance method for HRs and the Mantel-Haenszel method for RRs. Sensitivity analysis was performed by excluding the RECOVERY trial, which focused on patients with very severe AS. All statistical analyses were conducted using Review Manager (Version 5.4; Cochrane Collaboration).

The pooled analysis comprised 1426 patients from 4 RCTs.^7–10^ Baseline characteristics of the included patients are provided in Table 1. All included trials reported stroke incidences, with early intervention significantly reducing stroke occurrence by 39% compared with conservative care [RR: 0.61 (0.39–0.96); *P* = 0.03; *I*^2^ = 0%; Figure 1A]. Early intervention was associated with a significant 70% reduction in HHF [HR: 0.30 (0.18–0.51); *P* < 0.001; *I*^2^ = 0%; Figure 1B]. For mortality outcomes, all-cause and CV mortality showed no significant reductions with early intervention: all-cause mortality [HR: 0.75 (0.47–1.22); *P* = 0.25; *I*^2^ = 45%; Figure 1C] and CV mortality [HR: 0.78 (0.38–1.62); *P* = 0.51; *I*^2^ = 49%; Figure 1D]. Similarly, no effect was observed for SCD [RR: 1.10 (0.49–2.44); *P* = 0.82; *I*^2^ = 0%; Figure 1E] or MI [RR: 0.96 (0.27–3.41); *P* = 0.95; *I*^2^ = 0%; Figure 1F]. Sensitivity analysis excluding the RECOVERY trial revealed consistent results (see **Figures S1–S6**, **Supplemental Digital Content 1**, http://links.lww.com/AJT/A211).

**Table 1.** Baseline characteristics of included trials.

Trial characteristics	RECOVERY 2019		AVATAR 2021		EVOLVED 2024		EARLY TAVR 2024	
	Early	Conservative	Early	Conservative	Early	Conservative	Early	Conservative
Sample size (n)	72	72	78	79	113	111	455	446
Mean age (SD)	65.0 (7.8)	63.4 (10.7)	68 (7.6)	69 (7.9)	73.9 (8.2)	74.5 (9.0)	76.0 (6.0)	75.6 (6.0)
Median follow-up duration (yrs)	6.2		2.6		3.5		3.8	
Females, no. (%)	—	—	32 (41.0)	35 (44.3)	31 (27)	32 (29)	131 (28.8)	147 (33.0)
Medical history								
Body mass index, kg/m^2^ (SD)	24.7 (3.4)	24.0 (2.6)	27.4 (2.8)	27.9 (4.2)	27.5 (5.0)	27.9 (4.7)	28.4 (4.6)	28.6 (4.8)
Diabetes, no. (%)	13 (18)	7 (10)	14 (17.9)	23 (29.1)	15 (13)	26 (23)	119 (26.2)	114 (25.6)
Hypertension, no. (%)	40 (55)	39 (54)	69 (88.4)	70 (88.6)	76 (67)	70 (63)	369 (81.1)	365 (81.8)
Smoking, no. (%)	19 (26)	21 (29)	16 (20.5)	14 (17.7)	51 (45)	55 (50)	—	—
Previous PCI, no. (%)	3 (4)	1 (1)	1 (1.3)	2 (2.5)	7 (6)	7 (6)	—	—
Previous stroke, no. (%)	3 (4)	3 (4)	2 (2.5)	2 (2.5)	—	—	19 (4.2)	20 (4.5)
Peripheral vascular disease, no. (%)	1 (1)	2 (3)	—	—	4 (4)	9 (8)	33 (7.3)	21(4.7)
Coronary artery disease, no. (%)	5 (7)	1 (2)	1 (1.3)	3 (3.8)	—	—	133 (29.2)	113 (25.3)
Echocardiography								
Left ventricular ejection fraction, % (SD)	64.8 (5.2)	64.8 (4.1)	70.4 (8.3)	69 (9.1)	68 (9)	68 (8)	67.4 (6.5)	67.4 (6.7)
Peak aortic jet velocity, m/s (SD)	5.14 (0.52)	5.04 (0.44)	4.5 (0.4)	4.5 (0.3)	4.3 (0.5)	4.4 (0.5)	4.3 (0.5)	4.4 (0.4)
Aortic valve area, cm^2^ (SD)	0.63 (0.09)	0.64 (0.09)	0.67 (0.2)	0.75 (0.2)	0.8 (0.2)	0.8 (0.2)	0.9 (0.2)	0.8 (0.2)
Mean transaortic pressure gradient, mm Hg (SD)	64.3 (14.4)	62.7 (12.4)	51.3 (9.8)	50.2 (11.3)	45.2 (11.5)	45.0 (10.2)	46.5 (10.1)	47.3 (10.6)

AVATAR, aortic valve replacement versus conservative treatment in asymptomatic severe AS; EARLY TAVR, evaluation of TAVR compared with surveillance for patients with asymptomatic severe AS; EVOLVED, early valve replacement guided by biomarkers of left ventricular decompensation in asymptomatic patients with severe AS; PCI, percutaneous coronary intervention; RECOVERY, randomized comparison of early surgery versus conventional stenosis in very severe AS.

**FIGURE 1.**
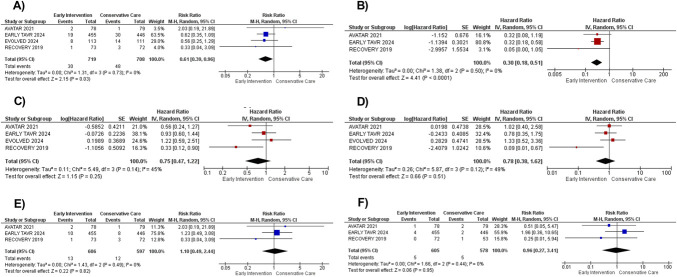
Forest plots for effect of early intervention on (A) stroke, (B) hospitalization for heart failure, (C) all-cause mortality, (D) CV mortality, (E) SCD, and (F) myocardial infarction among patients with asymptomatic AS.

This meta-analysis offers an updated, clinically relevant perspective on the benefits and limitations of early intervention in asymptomatic severe AS, supporting its role in reducing stroke risk and HHF while providing a nuanced view on mortality outcomes. By focusing exclusively on RCTs, our analysis overcomes many biases associated with observational studies, such as selection bias and uncontrolled confounders, that often lead to overestimated benefits. Importantly, although previous analyses suggested mortality benefits, our findings indicate no significant impact of early intervention on all-cause or CV mortality, emphasizing the need for judicious patient selection rather than broad implementation of early intervention. Furthermore, the low heterogeneity across trials (I^2^ < 50% for all outcomes) reinforces the consistency and robustness of our findings. By including only RCTs, this analysis reflects contemporary advancements in SAVR and TAVR, ensuring relevance to current clinical practice. Although early intervention can significantly reduce the incidence of stroke and HHF, the absence of a mortality benefit implies that early intervention should be targeted toward patients at a high risk of progression to symptomatic disease rather than applied uniformly. Clinically, these results advocate for a personalized approach to treatment, where factors such as peak aortic jet velocity, left ventricular remodeling, myocardial fibrosis, and other high-risk markers are used to identify patients who would benefit most from early intervention. For those without such high-risk markers, a conservative “watchful waiting” strategy may be equally effective in terms of mortality outcomes, avoiding the risks associated with invasive procedures.

Several limitations of this study warrant attention. First, as a study-level meta-analysis, our analysis lacks individual patient data, restricting our ability to conduct more granular, patient-specific analyses. Second, the mean age across studies was more than 63 years, limiting the generalizability of findings to younger populations who may have different risk profiles and disease progressions. Third, the limited availability of subgroup data for SAVR versus TAVR prevents in-depth analysis of potential differences between these interventions, an important aspect because outcomes may differ by procedure type.

In conclusion, this meta-analysis highlights the role of early intervention in reducing stroke incidence and HHF in patients with asymptomatic severe AS, although without a significant effect on all-cause mortality, CV mortality, and SCD. These findings underscore that early TAVR or SAVR may enhance quality of life by reducing progression to symptomatic AS among carefully selected patients. The lack of mortality benefit, however, calls for a more refined approach to patient selection, balancing clinical benefits against procedural risks. Future research should focus on meta-analyses with individual patient data and randomized studies that clearly stratify outcomes between TAVR and SAVR, enhancing risk assessment and optimizing intervention timing for asymptomatic severe AS.

## Supplementary Material
