# Supplementary material for: Should We Intervene Early in Asymptomatic Aortic Stenosis? Evidence From a Meta-Analysis
Source: Am J Ther. 2025 Feb 7;32(6):e581–3. doi: 10.1097/MJT.0000000000001881 (PMC12591542; doi:10.1097/MJT.0000000000001881)

**Figure S1.** Forest plot for stroke after excluding RECOVERY 2019.

**
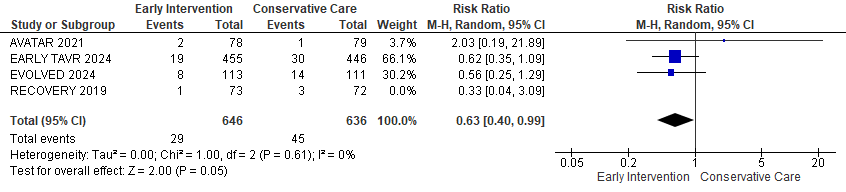
**

**Figure S2.** Forest plot for heart failure hospitalization after excluding RECOVERY 2019.


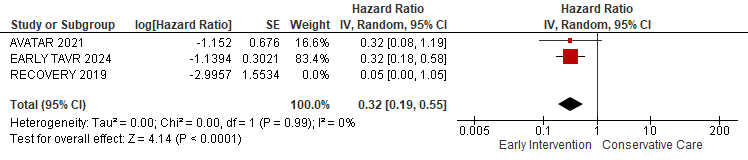


**Figure S3.** Forest plot for all-cause mortality after excluding RECOVERY 2019.


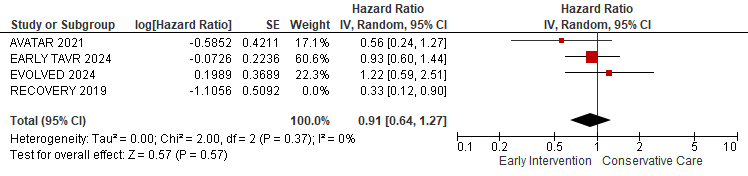


**Figure S4.** Forest plot for cardiovascular mortality after excluding RECOVERY 2019.


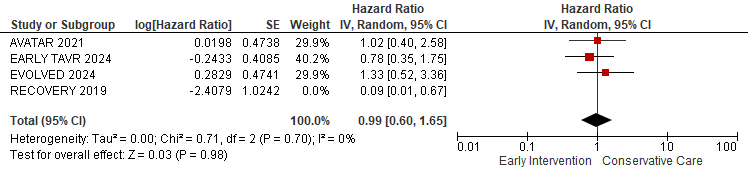


**Figure S5.** Forest plot for sudden cardiac death after excluding RECOVERY 2019.


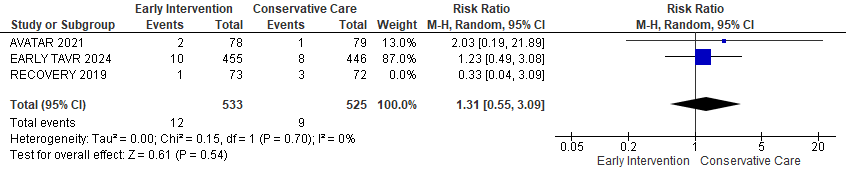


**Figure S5.** Forest plot for myocardial infarction after excluding RECOVERY 2019.


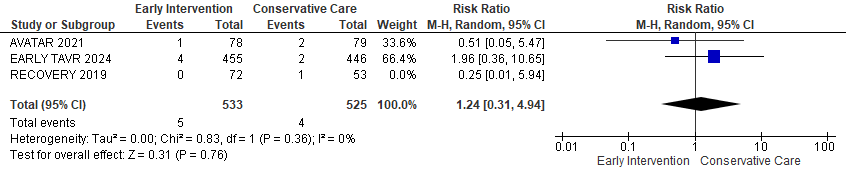

Supplement: Supplementary file 1 [file ajt-32-e581-s001.docx]
